# Le syndrome d'embolie graisseuse post traumatique

**DOI:** 10.11604/pamj.2014.17.83.2062

**Published:** 2014-02-02

**Authors:** Adnane Mohamed Berdai, Abdelkarim Shimi, Mohammed Khatouf

**Affiliations:** 1 Service de réanimation polyvalente A1; Centre hospitalier universitaire Hassan II, Fès, Maroc

**Keywords:** Syndrome d′embolie graisseuse, traumatisme, fracture des os longs, critères de Gurd, fat embolism syndrome, traumatism, long bone fractures, Gurd criteria

## Abstract

Le syndrome d'embolie graisseuse est une complication grave des fractures des os longs, il est la conséquence de la dissémination des particules graisseuses dans la microcirculation. L'objectif de ce travail est de déterminer le profil épidémiologique, la présentation clinique et paraclinique de ce syndrome et sa prise en charge thérapeutique. Notre étude porte sur 11 cas de syndrome d'embolie graisseuse colligés au service de réanimation A1 au centre hospitalier universitaire Hassan II de Fès, de Janvier 2009 à Juin 2012. Le diagnostic positif est basé sur les critères de Gurd. Les cas collectés se caractérisent par la prédominance du sexe masculin, d'un âge inférieur à 40 ans, présentant une fracture fémorale. Ce syndrome survient souvent dans les 72 heures après le traumatisme. La présentation clinique est dominée par l'hypoxémie et les troubles de conscience. Sur le plan biologique: l'anémie et la thrombopénie sont les manifestations les plus fréquentes. La prise en charge est symptomatique, 63% des patients ont nécessité l'intubation et la ventilation. L’évolution n'est pas toujours bénigne. Nos résultats confirme le polymorphisme de la présentation clinique et paraclinique du syndrome d'embolie graisseuse. Le diagnostic de ce syndrome se base sur des critères cliniques, mais reste essentiellement un diagnostic d’élimination. La prise en charge est symptomatique. La prévention de ce syndrome est essentielle et se base sur une fixation précoce des fractures des os longs.

## Introduction

Le syndrome d'embolie graisseuse (SEG) est une complication essentiellement liée à la traumatologie surtout en cas de fractures des os longs. C'est un ensemble de manifestations pulmonaires et systémiques secondaire à l'obstruction des petits vaisseaux par des particules graisseuses [1]. Son diagnostic reste sujet à controverse, du fait du polymorphisme des signes cliniques et de l'association fréquente à des traumatismes associés. Les critères de Gurd restent les plus utilisés pour le diagnostic positif de ce syndrome [2]. Le but de cette étude est de décrire les caractéristiques épidémiologiques, cliniques et thérapeutiques du SEG.

## Méthodes

C'est une étude rétrospective réalisée au service de réanimation A1 au niveau du centre hospitalier Hassan II à Fès, pendant la période allant de Janvier 2009 à Juin 2012. Sont inclus tous les patients présentant un SEG. Le diagnostic de syndrome est basé sur les critères de Gurd (Tableau 1) [2], il est retenu devant l'association d'un critère majeur et quatre critères mineurs ou bien de deux critères majeurs et deux critères mineurs à la suite d'un polytraumatisme ou d'un traumatisme des membres. Les données collectées incluait: les données démographiques, la durée d'intervalle libre, le mécanisme du traumatisme, la présentation clinique, les données biologiques et radiologiques, et enfin, la prise en charge thérapeutique et l’évolution.

**Tableau 1:** Les critères de Gurd pour le diagnostic du syndrome d'embolie graisseuse.

Critères majeurs	Critères mineurs
**Pétéchies**	Tachycardie > 120 battements/min
**Insuffisance respiratoire**	Fièvre
**Troubles neurologiques**	Anomalies rétiniennes: graisse ou pétéchie
	Ictère
	Signes rénaux: anurie ou oligurie
	Thrombopénie
	Anémie
	Elévation de la vitesse de sédimentation des érythrocytes
	Macroglobulinémie graisseuse

## Résultats

Onze cas de SEG ont été collectés dans notre centre formation pendant la période de l’étude. La fracture du fémur était présente chez 8 patients (72%), elle était bilatérale dans 2 cas et isolé dans un cas, le tibia est fracturé dans 4 cas et l'humérus chez 3 patients. 9 patients avaient de multiples fractures, alors que 2 avaient une fracture isolé l'un du fémur et l'autre de l'humérus. 4 patients avaient des traumatismes associées autres que celles des membres ou du bassin.

Les données démographiques montrent une prédominance des sujets jeunes males, en effet, dix (90%) patients étaient des hommes. L’âge moyen était de30,6 ans avec des extrêmes de 23 ans à 63 ans. Tous étaient victimes de traumatisme, le mécanisme le plus fréquent était les accidents de la voie publique dans 7 cas (63%), puis la chute d'un lieu élevé dans 2 cas, un cas de chute de sa hauteur et un cas d'impact direct par un bâton.

**Figure 1:**
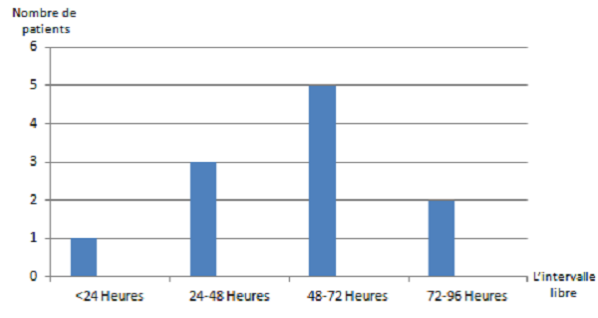
Durée de l'intervalle libre entre le traumatisme et l'apparition des symptômes du syndrome d'embolie graisseuse

Le délai moyen séparant le traumatisme du début des symptômes était de 54 heures, avec des extrêmes de 16 heures à 4 jours (Figure 1). Dans 7 cas, le SEG survenait en préopératoire avant la fixation chirurgicale, le foyer fracturé était stabilisé soit par une attelle ou par une traction selon le site fracturé. Dans 3 cas, le SEG est survenue en post opératoire, alors qu'il est survenu dans le cas restant en peropératoire au moment de l'alésage du tibia.

Sur le plan clinique (Tableau 2), 9 patients (81%) étaient hypoxémiques, deux d'entre eux ont développé un syndrome de détresse respiratoire aigu. Les signes neurologiques étaient présents dans 8 Cas (72%), tous avaient des troubles de conscience avec un Glascow coma scale (GCS) variant de 6 à 14, un patient avait présenté des crises convulsives généralisés tonico cloniques. Les pétéchies étaient observées chez 6 patients (54%), deux d'entre eux avaient des pétéchies conjonctivales.

**Tableau 2:** Les critères diagnostics du syndrome d'embolie graisseuse basés sur la classification de Gurd.

Signes cliniques et biologiques	Nombre de patients (%)
**Critères majeurs**	
Insuffisance respiratoire	9 (81%)
Troubles neurologiques	8 (72%)
Pétéchies	6 (54%)
**Critères mineurs**	
Tachycardie > 120 battements/min	6 (54%)
Anémie	8 (72%)
Thrombopénie	9 (81%)
Fièvre	6 (54%)

Concernant les signes mineurs, 6 cas (54%) ont présenté une tachycardie > 120 battements /mn, 8 (72%) une anémie inexpliqué, 9(81%) une thrombopénie< 150000 éléments/mm3 et 6 (54%) une fièvre > 39°C. on n'a pas effectué de recherches de lipurie ou de particules lipidiques dans le sérum.

La radiographie thoracique était réalisée chez tous les patients. Initialement, elle était normale dans 5 cas (45%). Elle a montré par la suite dans 9 cas (81%) un infiltrat alvéolo-interstitiel. La tomodensitométrie (TDM) cérébrale était normale dans 4 cas et a objectivé un oedème cérébral diffus dans un cas. L'imagerie par résonance magnétique (IRM) cérébrale était effectué chez un patient et était en faveur d'un SEG, elle montrait des plages en hypersignal T2 et diffusion intéressant la substance blanche du centre ovale, les deux capsules internes, les deux thalamus, le corps calleux, le noyau lenticulaire droit et le tronc cérébral.

La prise en charge était essentiellement symptomatique, incluant dans tous les cas une oxygénothérapie, un remplissage vasculaire et une analgésie adéquate. Une corticothérapie préventive a été administrée dés l'admission chez 4 patients, avec une bonne évolution chez 3 d'entre eux et le décès du quatrième. L'intubation et la ventilation artificielle étaient réalisées dans 7 cas (63%), elle était indiquée dans 4 cas devant une hypoxémie sévère, et dans les trois autres cas suite à une altération de la conscience (GCS<8).

La durée moyenne de ventilation était de 14 jours avec un maximum de 32 jours. Le décès est survenue dans 4 cas (36%), dont un suite à un choc septique, les 3 autres décès était secondaires aux conséquences du SEG (hypoxie sévère, forme fulminante). Un seul patient a gardé des séquelles neurologiques après six mois d’évolution.

## Discussion

Le SEG est un ensemble de manifestations cliniques consécutives à des embols de graisse. C'est une réaction inflammatoire systémique, essentiellement de la microcirculation pulmonaire, cérébrale et cutanée, secondaire à des embols de globules graisseux, issus d'une matrice osseuse rompue [3].

Gurd a proposé en 1970 des critères diagnostiques qui sont toujours utilisés, ils comportent des critères majeurs et des critères mineurs, le diagnostic de SEG est porté devant l'association d'un critère majeur et de 4 critères mineurs avec en plus la présence d'une macroglobinémie graisseuse [2]. La plupart des auteurs ont abandonné ce dernier critère, les globules graisseux peuvent être retrouvés aussi bien chez les traumatisés que chez les sujets sains [4, 5]. Un autre système diagnostic a été proposé par Schonfeld (Tableau 3) [6] ou des points sont attribués à différents critères diagnostiques, le diagnostic est retenu devant un score supérieur ou égale à 5, ce système à l'inconvénient d'exclure les patients présentant un traumatisme cérébral ou thoracique.

**Tableau 3:** Les critères de schonfeld pour le diagnostic du syndrome d'embolie graisseuse.

**Pétéchies**	5
Anomalie à la radiographie du thorax (Infiltrat alvéolaire diffus)	4
Hypoxémie (PaO2< 9.3 kPa)	3
Fièvre (38.8C)	1
Tachycardie (120 battements/ min)	1
Tachypnée (30 cycles/min)	1
Le score est calculé en additionnant les points donnés à chaque critère. 5 est le score requis pour le diagnostic.	

Le diagnostic du SEG est essentiellement clinique, la difficulté de ce diagnostic découle d'une part du fait que certaines manifestations comme l'hypoxémie ou les trouble de conscience sont communes en traumatologie à d'autres lésions associées, et d'autres part au caractère transitoire de certains signes cliniques. Le SEG reste avant tout un diagnostic d’élimination. La chronologie d'apparition des symptômes doit être évocatrice et la notion d'intervalle libre est primordiale. Nous avons retenu comme critères diagnostiques ceux proposés par Gurd ainsi que l'association de deux critères majeurs et deux critères mineurs de la même classification déjà utilisé par un Bulger et al [7].

L'incidence du SEG varie de 0,5 à 2,2% [8], cependant, il est difficile d’établir une incidence exacte de ce syndrome, elle peut varier de moins de 1% dans les séries rétrospectives à plus de 11% dans les études prospectives et en post mortem [9].

Le moment de la fixation de la fracture semble modifier l'incidence du SEG. Dans trois études rétrospectives [10–12] concernant la fracture du fémur, tous les cas de SEG étaient survenue dans le groupe ou il y avait une fixation chirurgicale retardée, et aucun cas n'est survenue lorsque la fixation était précoce, une étude prospective randomisée vient confirmer l'intérêt d'une fixation précoce lors de la fracture du fémur dans la prévention du SEG [13]. Dans notre série aucun patient n'avait bénéficié d'une fixation chirurgicale avant 72 heures, ce qui a exposé les patients aux risques de développer un SEG.

La littérature suggère une incidence élevée du SEG chez les patients présentant plus d'une fracture d'un os long [7, 14], nos données retrouve que 81% des patients ayant une SEG, ont plus d'une fracture d'un os long. L'os fracturé le plus incriminé dans la survenue du SEG est le fémur dans 60 à 92% des cas, suivi du tibia [7, 15, 16]. Dans notre série, 72% des patients avaient une fracture du fémur. Lorsque la fracture du fémur est associée à celle du tibia ipsilatéral, l'incidence du SEG augmente jusqu’à 13% [17].

Dans notre étude, 90% des patients étaient des males de moins de 45 ans, ceci est expliqué par le mécanisme lésionnel des traumatismes induisant le SEG. En effet, dans 63% des cas, il s'agissait d'accidents de la voie publique, vient ensuite les chutes d'un lieu élevé. La population exposée à ces accidents est dans notre contexte le male jeune.

Les manifestations respiratoires sont souvent les premiers signes à paraitre, parmi lesquelles, on retrouve: la tachypnée, la dyspnée et l'hypoxémie. Dans prés de la moitié des cas, l'atteinte respiratoire entraine une hypoxémie sévère associée à une insuffisance respiratoire recquiérant la ventilation artificielle [18]. L'atteinte respiratoire est la manifestation la plus fréquente dans notre série, elle est retrouvée chez 81% des cas, avec le développement d'un SDRA chez 18% des patients. La radiographie peut être normale au départ, en effet les gouttelettes lipidiques détachées de la médullaire osseuse, passant a travers les vaisseaux et atteint la circulation pulmonaire, cela crée un effet shunt entrainant l'apparition des symptômes respiratoires, dans les heures qui suivent, apparait une irritation du parenchyme pulmonaire, entrainant la libération de toxines, le poumon est alors infiltré et des images typiques d'oedème pulmonaire peuvent être retrouvé sur la radiographie du thorax[16, 19]. Dans notre série, on a constaté chez 3 patients un décalage entre la symptomatologie respiratoire et l'imagerie thoracique. Par la suite, tous les patients ont présenté un syndrome alvéolaire ou interstitiel à la radiographie thoracique.

Les manifestations neurologiques étaient présentes chez 72% des patients, essentiellement sous forme de trouble de conscience, et dans un cas des crises convulsives. Elles ne sont rattachées au SEG qu'après avoir éliminé une autre étiologie d'atteinte neurologique: traumatique ou infectieuse. Leur incidence varie dans la littérature entre 59% et 76% [7, 16]. Elles se manifestent par des troubles de la vigilance, allant d'une agitation à un coma profond. L'examen neurologique est souvent fluctuant et peut retrouver des signes neurologiques peu spécifiques (nystagmus, hypertonie extrapyramidale). Les signes déficitaires sont habituellement peu fréquents et habituellement transitoires. Des troubles neurovégétatifs (tachycardie, sueurs, hyperthermie) sont possibles, en relation avec une atteinte des noyaux gris centraux [1]. L'imagerie occupe une place importante dans le diagnostic positif de l'atteinte neurologique dans le SEG. La TDM cérébrale souvent de première intention dans un contexte traumatique permet d’éliminer une urgence neurochirurgicale, et met parfois en évidence des hypodensités localisées, un oedème cérébral diffus, mais dans la grande majorité des cas, la TDM cérébrale est normale [20, 21]. Dans notre série, malgré une atteinte neurologique clinique, la TDM cérébrale était normale dans 4 cas et a objectivé un oedème cérébral chez un seul patient, l'IRM présente un intérêt particulier dans le diagnostic du SEG, elle peut mettre en évidence des anomalies diffuses, sous forme d'hyposignal en séquences pondérées T1 et en hypersignal en séquences pondérées T2 [20]. L'analyse spectrophotométrique peut être intéressante en montrant des corps lipidiques au sein des lésions observées [1]. L'IRM était réalisée chez un patient et a montré des signes en faveur d'un SEG.

Le purpura pétéchial est retrouvé dans prés de 40% des cas, longtemps considéré comme pathognomonique, il est inconstant et sans valeur pronostique [22]. Il peut être le dernier élément de la triade à apparaitre. Il est dû à l'embolisation des petits capillaires au niveau du derme, entrainant une extravasation des érythrocytes. Il se localise habituellement au niveau des conjonctives, de la muqueuse orale et la région axillaire et cervicale [18]. Dans notre série, il n'est présent au niveau des conjonctives que dans deux cas.

D'autres manifestations peuvent être présentes, une rétinopathie se manifestant par des hémorragies rétiniennes, des taches blanches cotonneuses au voisinage des vaisseaux rétiniens ou par un oedème de la macula. Des atteintes rénales ou myocardiques sont aussi rapportés [1].

Sur le plan biologique, la thrombopénie est l’élément le plus constant, d′intensité variable, s′intégrant parfois dans un tableau de coagulation intraveineuse disséminé. L'anémie hémolytique est fréquente [23]. Cependant, aucun test biologique n'est spécifique, les dosages de la C réactive protéine, la recherche des particules lipidiques intracellulaires dans les prélèvements trachéaux ou dans le lavage broncho-alvéolaires ne sont ni sensibles, ni spécifiques [20]. Dans notre série la thrombopénie et l'anémie étaient les anomalies biologiques les plus rencontrées avec respectivement 81% et 72% des cas.

La prise en charge du SEG est, avant tout, préventive, visant à limiter l'importance quantitative des emboles graisseux, elle se base sur la gestion du stress, l'analgésie, l'oxygénothérapie, le remplissage vasculaire et la réduction-immobilisation des fractures [24]. La fixation chirurgicale doit être précoce dans les 24 heures après une fracture d'un os long [25]. L'intérêt d'une corticothérapie à titre préventif n'est pas confirmé, les corticostéroïdes peuvent aider à prévenir l'hypoxie, mais n'améliorent pas la mortalité [26]. Par ailleurs, ils présentent des contre-indications chez les patients ayant subi un traumatisme grave ou en période de soins intensifs [5]. Seulement 4 patients ont reçu une corticothérapie, dont 3 ont présenté une évolution favorable, des conclusions ne peuvent être tirées de ce nombre limité de cas.

La prise en charge thérapeutique chez nos patients était essentiellement symptomatique, basée sur l'oxygénothérapie, un remplissage vasculaire assurant une volémie correcte, et une analgésie adéquate. L'intubation était indiquée chez 7 (63%) patients, 4 sur des critères respiratoires et 3 suite à une altération neurologique, ce taux varie entre 44% et 75% selon les différentes séries [7, 15, 16], la durée moyenne de ventilation était de 14 jours. En cas d'oedème cérébral secondaire à l′embolie graisseuse ou d'un traumatisme crânien associé; les niveaux de la pression positive de fin d′expiration était revue à la baisse.

La prise en charge chirurgicale de ces patients consistait en un enclouage centromédullaire des fractures du fémur et du tibia et la mise en place d'une plaque vissée dans les fractures de l'humérus. En cas d'enclouage centromédullaire, il est recommandé de réaliser un trou cortical de ventilation, d'effectuer un lavage abondant ou un drainage aspiratif de la cavité et de supprimer les alésages [22].

Un traitement spécifique de la SEG n'existe pas actuellement, des traitements avec de l'héparine, Dextran, et les corticostéroïdes n'ont pas démontré de diminution de la mortalité ou de la morbidité [27], le traitement du SEG est donc uniquement symptomatique basé sur la réanimation cardio respiratoire, l'oxygénothérapie doit avoir comme objectif une PaO2 supérieure à 90 mmhg, une volémie correcte doit être assurée afin d’éviter l'aggravation des lésions pulmonaires causées par les embols de graisse [28]. Le remplissage par de l'albumine pourra être bénéfique, il se lie par une liaison covalente avec les acides gras libres, ce qui diminuerait leur toxicité potentielle [22].

La mortalité est associée à la sévérité du SEG et aux lésions associées, dans notre série la mortalité était de 36% (4 cas), 3 décès étaient secondaires aux conséquences directs du SEG (hypoxie sévère, forme fulminante). Le pronostic final dépend de l′atteinte pulmonaire et neurologique, Les progrès de la réanimation ont permis une meilleure prise en charge du syndrome de détresse respiratoire aigu et de diminuer ainsi la mortalité des formes graves. La guérison survient en une quinzaine de jours. Lorsqu′elle est obtenue, elle est le plus souvent totale, Néanmoins, des séquelles pulmonaires et surtout des désordres psychiatriques, déficitaires ou comitiaux ont été rapportés [23]. Dans notre série, un patient a gardé des séquelles neurologiques après 6 mois d’évolution. Sur le plan neurologique, L'IRM pourrait être un indicateur pronostic en différenciant entre l'oedème vasogénique et des lésions plus sévères, l'existence d'un foramen ovale semble être associée à des lésions cérébrales graves [5].

## Conclusion

Nos résultats confirment la variabilité de la présentation et de l’évolution du SEG. Le diagnostic se base sur un faisceau d'arguments, mais reste essentiellement un diagnostic d’élimination. Bien que le SEG ait un impact sur la survie, La prise en charge reste symptomatique nécessitant parfois une ventilation artificielle. La fixation précoce des fractures des os longs reste le meilleur moyen de prévention de ce syndrome.
